# Harnessing Molecular Fluorophores in the Carbon Dots Matrix: The Case of Safranin O

**DOI:** 10.3390/nano12142351

**Published:** 2022-07-09

**Authors:** Manuela Meloni, Luigi Stagi, Davide Sanna, Sebastiano Garroni, Laura Calvillo, Angela Terracina, Marco Cannas, Fabrizio Messina, Carlo Maria Carbonaro, Plinio Innocenzi, Luca Malfatti

**Affiliations:** 1 Laboratory of Materials Science and Nanotechnology (LMNT), Department of Biomedical Sciences, University of Sassari, 07100 Sassari, Italy; manmeloni@uniss.it (M.M.); dvdsanna@uniss.it (D.S.); 2 Department of Chemistry, Physics, Mathematics and Natural Sciences, University of Sassari, 07100 Sassari, Italy; lstagi@uniss.it (L.S.); sgarroni@uniss.it (S.G.); 3 Department of Chemical Sciences, University of Padua, 35131 Padua, Italy; laura.calvillolamana@unipd.it; 4 Department of Physics and Chemistry Emilio Segrè, University of Palermo, 90123 Palermo, Italy; angela.terracina@unipa.it (A.T.); marco.cannas@unipa.it (M.C.); fabrizio.messina@unipa.it (F.M.); 5 Department of Physics, University of Cagliari, Cittadella Universitaria, 09042 Monserrato, Italy; cm.carbonaro@dsf.unica.it

**Keywords:** carbon dots, safranin, phosphors, nanoparticles

## Abstract

The origin of fluorescence in carbon dots (C-dots) is still a puzzling phenomenon. The emission is, in most of the cases, due to molecular fluorophores formed in situ during the synthesis. The carbonization during C-dots processing does not allow, however, a fine control of the properties and makes finding the source of the fluorescence a challenging task. In this work, we present a strategy to embed a pre-formed fluorescent molecule, safranin O dye, into an amorphous carbonaceous dot obtained by citric acid carbonization. The dye is introduced in the melted solution of citric acid and after pyrolysis remains incorporated in a carbonaceous matrix to form red-emitting C-dots that are strongly resistant to photobleaching. Embedding dyes in amorphous C-dots represents an alternative method to optimize the emission in the whole visible spectrum.

## 1. Introduction

Carbon dots (C-dots) are generally defined as quasi-spherical carbon-based nanomaterials with a particle size smaller than 10 nm showing remarkable photoluminescence properties [1,2,3,4]. Up to now, even if an exceptional number of articles has been published about the structural properties of C-dots, the origin of their fluorescence is still under debate [5]. Several models have been proposed to describe their peculiar properties; one of the most popular considers the C-dots as a carbonaceous matrix that incorporates molecular fluorophores. The fluorescence is originated by n–π* and π–π* charge transfer transitions within the fluorophores or through a strong interaction with the matrix [6,7]. These fluorophores form via carbonization of nitrogen- or oxygen-rich organic molecules [8,9]. A complete control of the emission is hard to achieve because of the different reaction pathways followed by the precursors during the possible processing routes, solvothermal and microwave treatments, or pyrolysis. The photoluminescence is the result of combined emissions from fluorophores of different structures and composition, from single molecules to fluorescent conjugate polymers. Moreover, the lack of an adequate description of the C-dot structure and the low reproducibility make it difficult to optimize the emission. An alternative to the “conventional” methods is embedding molecular fluorophores into carbonaceous particles of nanometric size. Citrazinic acid, for instance, is as a fluorophore capable of providing a strong emission in the blue when it is used as a C-dot precursor [10]. On the contrary, citrazinic acid itself shows a small quantum yield (QY) and a strong tendency to form non-fluorescent aggregates, even at low concentrations [11]. In addition, a carbon matrix embedding molecular fluorophores provides good biocompatibility and endless possibilities of chemical functionalization of the surface. The advantage offered by the incorporation of fluorescent molecules into a C-dot structure has been neglected up to now, and most of the literature focuses on the formation of specific C-dots with tailored emission properties and high QY.

In this work, we have used C-dots as a matrix to embed fluorophores with known properties, to take advantage of the chemical passivation and protection offered by the carbon nanoparticles. In particular, we have specifically selected a chromophore emitting in the red region of the visible spectrum. One of the main challenges for C-dots, in fact, is achieving intense emissions in the red and infra-red regions. The reproducibility and stability of these spectral properties would allow the creation of reliable white light-emitting diodes and envisage breakthrough applications in biomedical theranostics, biosensing, and bioimaging [12]. For these reasons, several efforts have been dedicated to the synthesis and applications of red-emitting C-dots.

Several attempts to prepare red C-dots starting from red fluorophores, with the purpose of exploiting the potentiality of such an approach, have been reported so far. Some examples of carbon structures bearing dye molecules entrapped within the core have already been reported. The nanoparticles, however, have redshifted emission and broader adsorption in comparison with the corresponding free dyes [13]. Red/near infrared (NIR) emitting C-dots have been prepared using a hydrophobic cyanine dye (CyOH) and polyethylene glycol. The resulting C-dots have high photothermal conversion efficiency and higher hydrophilicity compared with CyOH itself [14]. Furthermore, red-emitting C-dots via one-pot hydrothermal synthesis from citric acid (CA) and neutral red (NR) have proved to be effective in the optical detection of noble metal ions in PC12 cells and zebrafish [15].

In the present work, we have obtained red-emissive and photoresistant C-dots using one of the most common red fluorophores, Safranin O (SO). This chromophore is extensively used in histology and cytology as a biological stain [16,17]. Safranin O itself, while it exhibits a red emission in solution, does not emit in the solid state as with the majority of organic dyes. Furthermore, the dye emission undergoes severe photobleaching under laser light. For these reasons, red-emitting C-dots with a high resistance to photobleaching represents a valuable nanomaterial. The incorporation of Safranin O into a carbonaceous matrix allows the extending of its field of application into the solid state.

## 2. Materials and Methods

### 2.1. Materials

Safranin O, or 3,7-diamino-2,8-dimethyl-5 phenylphenazinium chloride, (SO dye, Sigma-Aldrich (Milan, Italy) and citric acid monohydrate (CA, Fluka (Milan, Italy)), >99.5%) were used as received without further purification. Milli-Q water was used for the analysis.

### 2.2. Synthesis and Purification of Red C-Dots

A total of 3.84 g of CA (0.02 mol) were placed into a 50 mL round flask in its crystalline form and dipped into a preheated 200 °C oil bath (with a stirring rate of 350 rpm using a Teflon-coated stirring bar). After that, 0.35 g of SO (0.001 mol) were added when the CA was completely melted. Samples were taken at three determined reaction times for analysis: CD0 (0 min), CD2.5 (2.5 min), and CD15 (15 min). The obtained reaction products result in a dark red precipitate. Afterwards, they were dispersed in Milli-Q water and purified through dialysis bags (cut off ~14,000 Da), replacing the water every 12 h to remove unreacted molecules. Finally, the C-dot solutions were dried at 60 °C for 12 h into an oven in air to obtain the solid powders.

### 2.3. Characterization Techniques

The C-dot composition was determined by X-ray photoelectron spectroscopy (XPS) using a custom-designed UHV system equipped with an Omicron electron analyzer, working at a base pressure of 10^−10^ mbar. A non-monochromatized Al Kα X-ray source (1476.6 eV) was used to acquire the core-level photoemission spectra. Single spectral regions were collected using 0.1 eV steps, 0.5 s collection time, and 20 eV pass energy. The C 1s region was separated into five chemically shifted components, although not all of them are present in all the samples. The Doniach–Sunjic shape was used for the sp^2^ component, whereas symmetrical Voight functions were used for the sp^3^ component and the C-O and C-N functional groups. In the case of the N 1s region, four chemically shifted components were considered, and symmetrical Voight functions were used for all of them. The same full width at half maximum (FWHM) was used for all the components in the same region.

Infrared (IR) absorption measurements were performed on powder samples. The spectra were collected by the use of a Bruker Vertex 70 spectrometer in ATR mode in the range 4000–400 cm^−1^ with 32 scans and a resolution of 4 cm^−1^.

UV-Vis absorption spectra were recorded in absorbance mode by the Nicolet Evolution 300 spectrophotometer from 200 to 600 nm.

Fluorescence spectroscopy measurements were carried out using a Horiba Jobin Yvon (Rome, Italy) Fluoromax-3. The 3D maps were recorded in the 300–700 nm interval using water-dispersed samples. Photoluminescence QY measurements have been performed using the quanta-ϕ (HORIBA) integrating sphere accessory, attached to the “NanoLog” Horiba Jobin Yvon spectrofluorometer.

Time-resolved photoluminescence (TR-PL) measurements were performed by exciting the samples with 200 fs long pulses delivered by an optical parametric amplifier (Light Conversion TOPAS-C), pumped by a regenerative Ti:sapphire amplifier (Coherent Libra-HE). The repetition frequency was 1 kHz, and the PL signal was recovered by a streak camera (Hamamatsu, Milan, Italy, C10910) equipped with a grating spectrometer Acton SpectraPro SP-2300 (Princeton Instruments, Trenton, NJ, USA).

Fluorescence loss in photobleaching (FLIP) experiments were performed by monitoring the changes of C-dot fluorescence when exposing C-dot samples to a series of high-intensity laser pulses (pls). A single laser beam served both as a source of photobleaching and to excite the fluorescence used to inspect the progress of the experiment, which was monitored by dispersing the light emitted by the sample on an i-CCD camera PI_Max (Princeton Instruments, Trenton, NJ, USA,). The progressive bleach of the fluorescence was investigated in the range of 0–10^5^ pulses (about 1 h) by collecting 10 pulses per spectrum. The beam was obtained through a pulsed tunable laser (Opotex Vibrant, Carlsbad, CA, USA) with a 10 Hz pulse frequency, approximately 5 ns pulse width. We carried out two types of FLIP experiments, under exposure to wavelengths of 532 nm (10 mJ/pulse) or 260 nm (0.15 mJ/pulse), respectively. All the experiments were performed by using 40 μL of samples in a 1 mm quartz cuvette and irradiating the entire volume. A further FLIP experiment was carried out by using a wavelength of 532 nm and a power of 0.15 mJ/pulse, but no fluorescence loss was observed. For this reason, the data are not shown.

Quantum yields were measured by inserting the samples in an integrating sphere (Labspehere 3p-gps-060sf, ig) and collecting their emission under excitation with laser diodes at the appropriate wavelengths.

Transmission electron microscopy images were obtained with FEI (Eindhoven, Netherlands) TECNAI 200 microscope operating at 200 kV. At first, C-dots were dispersed in ethanol, then a droplet of the solution was cast on a carbon-coated copper grid and let dry before the measurement. C-dot size was calculated from TEM images using ImageJ program.

TG measurements were carried out using SDT Q 600 TA INSTRUMENTS (Milan, Italy) by heating samples from 25 to 900 °C with a heating rate of 20 °C/min in a nitrogen atmosphere (20 mL/min).

DSC measurements were carried out to simulate the synthesis procedure by heating samples from 25 to 200 °C with a heating rate of 20 °C min^−1^. The final temperature was kept for 10 min and the heat-flow variation was evaluated in the function of reaction time.

## 3. Results and Discussion

The first step in obtaining red-emitting C-dots was to add safranin O to the molten citric acid. This process has the advantage of allowing the safranin O to be dissolved in a liquid phase and thus obtaining a more homogeneous dispersion and also facilitating the insertion of the dye into the matrix. Once exceeded the melting-point temperature, 150 °C, CA dehydrates and polymerizes to form a carbonized structure whose carbonization degree is controlled through temperature and reaction time [18]. On the contrary, the higher thermal stability of the dye preserves SO from thermal degradation up to 300 °C (see Figure S1 in Supplementary Information with TGA and DSC of CA, SO, and the combination of the two reagents)

**Figure 1:**
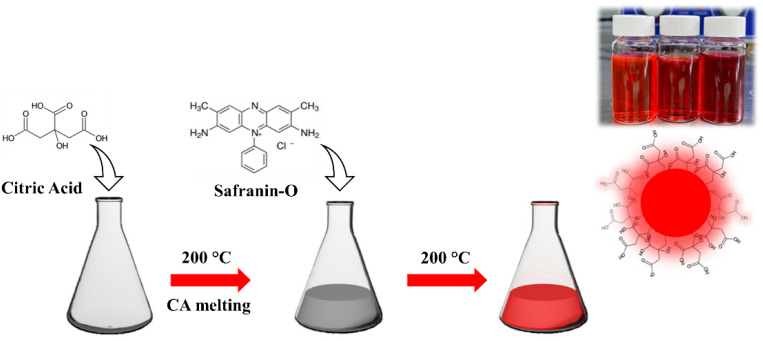
Scheme of C-dot synthesis.

Compared with other methods, such as microwave and solvothermal, the oil bath route has the advantage to allow for an easy sampling of the products as a function of the reaction time. We have used a temperature of 200 °C to assure the complete melting of CA and kept it constant throughout the experiment. A small amount of product (<100 μL) has been collected to monitor the progress of the thermal reaction from 0 up to 15 min. Figure 1 shows the Safranin-derived dot synthesis.

**Figure 2:**
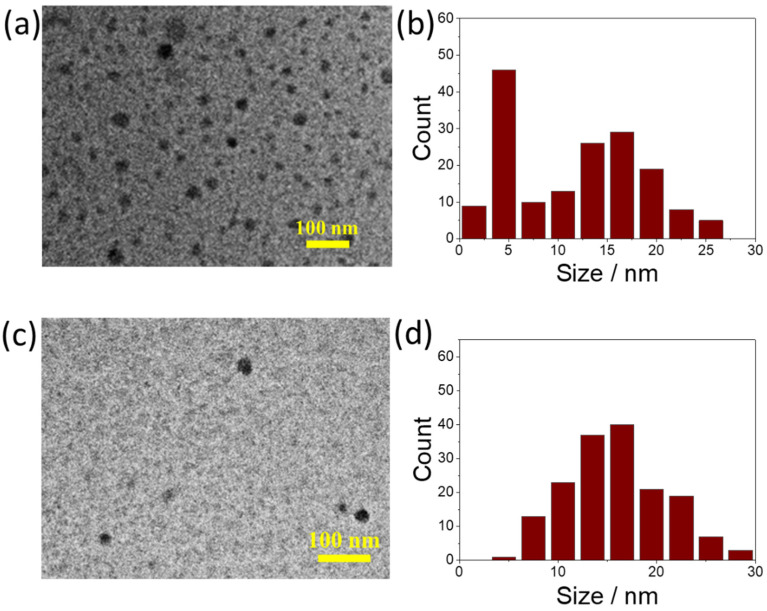
Representative TEM images of C-dots acquired at zero min (**a**) and 15 min (**c**) at 200 °C and corresponding size distribution of C-dots (**b**,**d**).

The morphology and the size of the as-prepared C-dots have been analyzed by transmission electron microscopy (TEM). TEM images in Figure 2a,c show that round-shaped nanoparticles are the final product; the particle size does not change until 15 min of reaction time is exceeded. The C-dots exhibit a size distribution from 5 to 25 nm (Figure 2b,d) with an average value of 15 ± 5 nm. There is no evidence of crystalline phases, even in dark field mode and in high resolution (not shown in the figure), suggesting that only amorphous C-dots have been formed.

**Figure 3:**
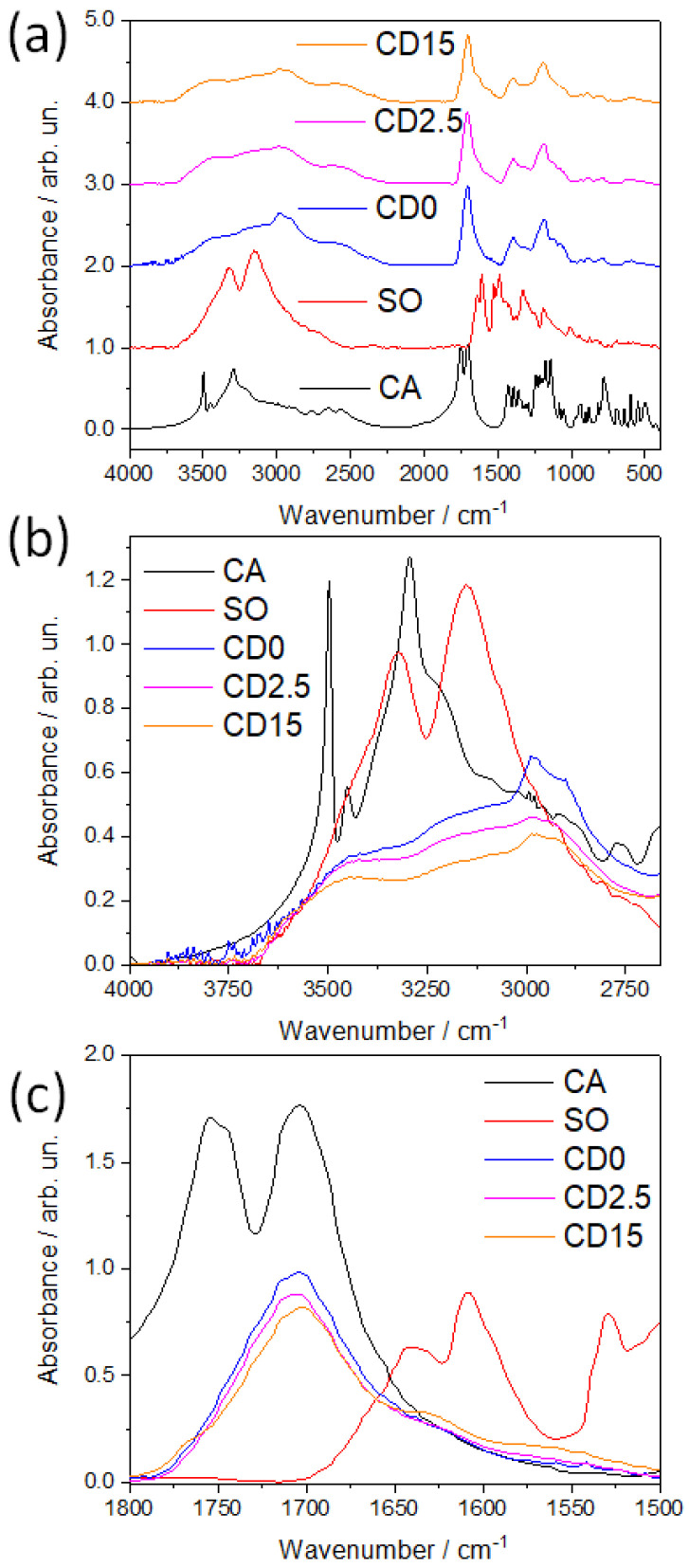
(**a**) FTIR absorption spectra of the CA and SO precursors and C-dots at 0-, 2.5-, and 15-min reaction times. (**b**,**c**) Enlargements of the IR spectral regions at 3600–3000 cm^−1^ and 1800–1550 cm^−1^.

FTIR absorption spectroscopy has been used to investigate the structural change induced by increasing the reaction time. Figure 3a shows the FTIR absorption spectra of five different compounds: CA and SO precursors and C-dots sampled at three reaction times (CD0 (0 min), CD2.5 (2.5 min), and CD15 (15 min)). The main bands of SO displayed at 3150 and 3325 cm^−1^ are attributed to the N-H symmetric and asymmetric stretching vibrations [19]. C-H stretching of the SO methyl group is located in the region between 2800 and 3000 cm^−1^ and contributes to the enlargement of high-frequency bands towards lower wavenumbers (Figure 3b). In the same spectral range, CA exhibits two characteristic bands at 3498 cm^−1^ and 3295 cm^−1^ along with minor contributions at 3222 and 3450 cm^−1^ (Figure 3b) that are assigned to the OH stretching from carboxylic groups [20]. Two broad bands at 3158 and 3450 cm^−1^ characterize the vibrational structure of the C-dots and are due to the large amount of OH groups formed in the pyrolyzed carbon structure and the presence of NH groups stretching, respectively. In addition, CH stretching is recognizable in the peaks at 2906 and 2980 cm^−1^ whose intensity decreases with the reaction time. In the region between 1500 and 1800 cm^−1^, SO shows two main peaks at 1641 and 1609 cm^−1^ that are correlated to in-plane H-N-H and C=C stretching in phenazine rings [21]. The other band with a maximum at 1529 cm^−1^ still stems from C=C stretching vibration in phenazine rings (Figure 3c). CA, in its anhydrous form, displays a doublet at 1754 and 1744 cm^−1^ and a further band at approximately 1703 cm^−1^ originating from the C=O stretching mode of the carboxyl group.

As a result of the thermal treatments, the doublet in C-dots disappears whilst the band at 1703 cm^−1^ is preserved for all the 15 min of the reaction. It can be assigned to the C=O stretching mode of carbonyl anhydride in the itaconic acid, an intermediate compound of the dehydration process of CA [22,23] and a well-documented structure in graphene carbon dots derived from CA [24]. The bands at 1636 and 1561 cm^−1^ that strengthen with the reaction time are compatible with C=O stretching and NH bending modes of amide I and amide II, respectively. A detailed analysis through band deconvolution is shown in Figure S2.

According to FTIR data, the thermal treatment of a mixture of CA and SO forms C-dots with a structure embedding or wrapping the dye molecules. The presence in the infrared spectra of the characteristic vibrational modes of the amide demonstrates the incorporation of SO in the carbonaceous structure and the interconnection between the two chemical species.

**Figure 4:**
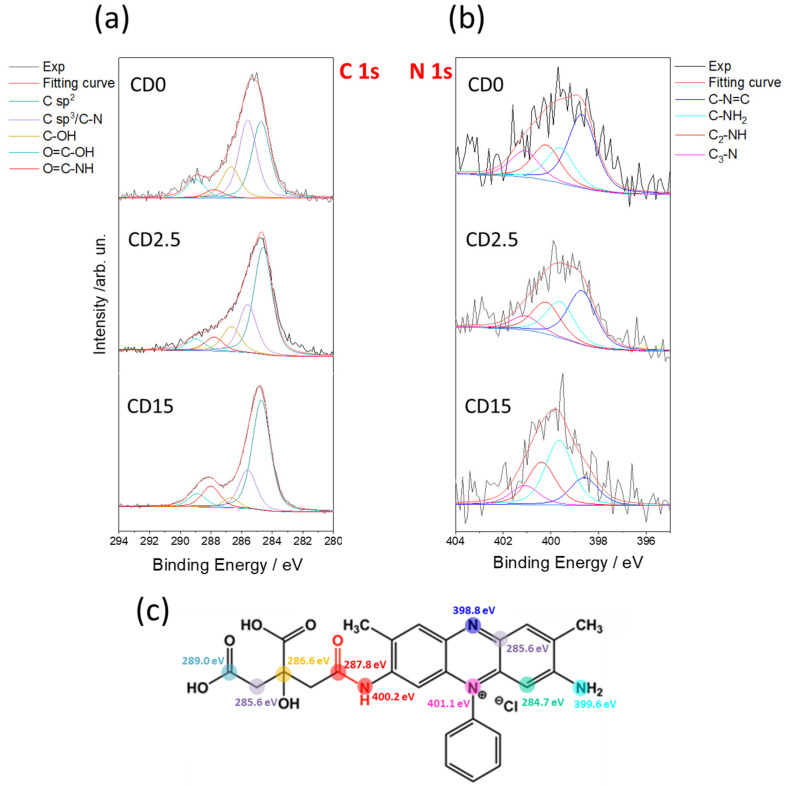
X-ray photoelectron spectroscopy (XPS) analysis of the C-dots at different reaction times. (**a**,**b**) C 1s and N 1s photoemission spectra and their deconvolution into single chemical components. (**c**) Association of the different expected C 1s and N 1s components used in the fit of the XPS spectra.

XPS analysis confirms the formation of characteristic bonds between SO and the C-dot matrix. The C 1s spectrum spectra in Figure 4 have been deconvoluted into five components corresponding to different hybridization states and chemical bonds, namely C sp^2^ (284.7 eV), C sp^3^/C-N (285.6 eV), C-OH (286.6 eV), C=O-NH (287.9 eV), and -COOH (288.9 eV). Table S1 and Figure S3 in the Supporting Information reports the spectra of CA and SO alone and all the resulting fitting parameters. The sp^2^ component of the C-dots gradually increases with the reaction time. The component is associated with an increase of SO embedded into the C-dot matrix with the proceed of the reaction. At the same time, the fraction of sp^3^ carbons reduces in accordance with the dehydration occurring in CA and the formation of π bond in a more disordered framework. The amount of C=O-OH groups also decreases with the reaction time, which can be considered as an indicator of the condensation between the carboxyl group of CA and the amino group of SO. This reaction forms the amide bonds, in accordance with FTIR data. The amide formation is also supported by the C=O-NH component that is detected at the beginning of the reaction as well as after 15 min. Such a condensation reaction is the key step to incorporate SO into the carbonaceous matrix formed upon the pyrolysis of CA.

Figure 4b provides some clues about the effect of pyrolysis on the N 1s lines. The deconvolution results into four components: C=N-C (398.8 eV), C-NH_2_ (399.6 eV), C_2_-NH (400.2 eV), and C_3_-N (401.0 eV). The reaction reduces the C=N-C component from 43.3% to 18.4% and conversely enhances the C_2_-NH from 20.9% to 25.6%, confirming the formation of the amide bond. C_3_-N remains stable throughout the reaction as the pyrimidine moiety of the SO remains also stable. Figure 4c resumes the expected components of C 1s and N 1s used to fit the XPS spectra.

**Figure 5:**
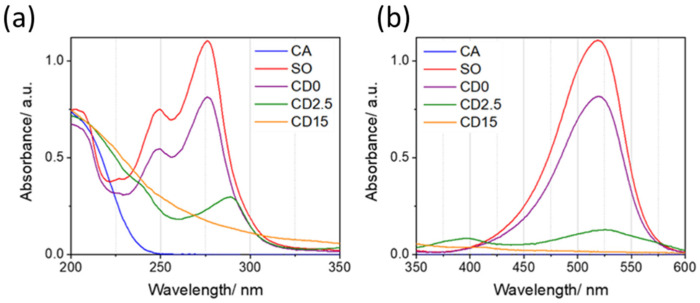
UV-Vis absorption spectra of CA, SO [0.01 mg mL^−1^], and C-dots obtained from the thermal decomposition of CA and SO at different reaction times; (**a**) 200–350 nm and (**b**) 350–600 nm range.

**Figure 6:**
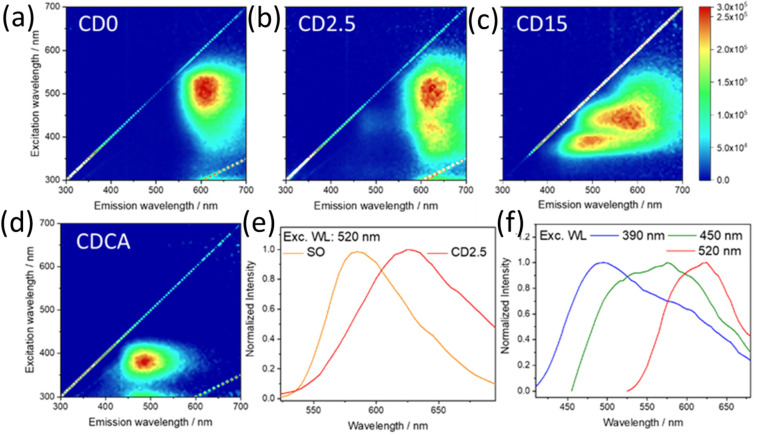
3D fluorescence spectra (emission (x-scale), excitation (y-scale), intensity (false colour scale)) of C-dots at different reaction times: 0 (**a**), 2.5 (**b**), and 15 min (**c**), respectively. The 3D fluorescence spectra of C-dots by CA only (CDCA), after 15 min of reaction time (**d**). Normalized photoluminescence spectra of SO and CD2.5 under excitation at 520 nm (**e**). Normalized photoluminescence spectra of CD15 as a function of excitation wavelength (390, 450, 520 nm) (**f**).

Figure 5 shows the UV-Vis absorption spectra of CA, SO, and C-dots after reaction at different times. The spectra are shown, for the sake of clarity, in two wavelength ranges: 200–350 (Figure 6a) and 350–600 nm (Figure 6b). CA shows one intense band at high energy (typically <250 nm) due to the characteristic π → π* electronic transition in the carbonyl group and no other absorption bands. This spectral feature is clearly shared with SO and C-dots as a result of transitions in the conjugate states of benzene moieties and aromatic states, respectively. SO π–π* transitions also extend to 248 and 276 nm bands [23]. Another absorption band (Figure 6b) in SO is centered around 520 nm and is attributed to intramolecular charge-transfer (ICT) n → π* transitions involving phenazine nitrogens [25,26,27].

At the beginning of the thermal reactions, CD0 shows an absorption spectrum that is very similar to pure SO. After 2.5 min, a new band arises at 241, 289, 400, and 527 nm. In the 15-min reacted compound, SO absorption fingerprints disappear, leaving an unstructured band ranging from 200 up to 500 nm. CD15 spectrum is comparable with the carbon dot spectra obtained by Dong et al. by CA polymerization at high temperature and correlated with the formation of an extended sp^3^ matrix and small sp^2^ domains within the sp^3^ matrix [28].

Figure 6a–d shows the 3D excitation (*y*-axis)-emission (*x*-axis)-intensity (normalized false color scale) spectra of the CD0, CD2.5, and CD15 dots, together with the CA derived C-dot. Figure 6e,f are the corresponding emission spectra of C-dots acquired using C-dot dispersions in water. CD0 exhibits the characteristic photoluminescence of SO with a broad band peaking at 595 nm, λ_ex_ = 520 nm. The emission is very close to that of pure SO (Figure S4) with a slight shift (~10 nm). This is attributed to the integration of SO in the carbonized polymeric CA, as the formation of the chemical bonds between the dye and the matrix affects the energy gap of the red-emitting transition of SO. After 2.5 min (Figure 6b), a further shift in emission, up to 615 nm, can be observed under the same excitation wavelength. The emission shift is clearly visible in the 2D spectra, under excitation at 520 nm (Figure 6e). At longer reaction times, no further shift of the red component has been observed, indicating that the reaction between the SO and CA has been accomplished and the dye is in a stable chemical environment provided by the C-dot structure.

Figure 6c shows the effect of 15 min of thermal reaction on the PL spectra. At this stage, a multiplicity of components stemming from different contributions overlaps SO red emission as a result of the progressive structural modulation of the carbonaceous matrix.

The rise of such a widely tunable blue-green emission in CD15 is due to the formation of a range of different fluorophores produced by CA pyrolysis. However, this is only observed in the presence of SO. In fact, the thermal treatment of CA alone only provides C-dots with a strong, spectrally isolated, blue emission (Figure 6d). While the formation of CA derived C-dots is well documented and will not be addressed in this work [18,29], it is worth mentioning that CD15 optical properties are not a mere summation of CA-based C-dots and SO characteristic emission. If the blue component is compatible with the carbonized CA structure, the large component in the green and yellow region results from the interaction with SO dye according to the modalities described in the structural characterization section. Furthermore, it is still possible to selectively excite at 520 nm the characteristic emission of bound SO, demonstrating that the fluorophores are still active after 15 min of reaction, although to a lesser extent (Figure 6f).

Based on the results of Figure 6, for further investigations, we focused on the CD2.5 sample where the reaction has proceeded to enough an extent that the SO moiety has been fully incorporated into a well-formed carbonaceous matrix, but the spectroscopic identity of the SO emitters is still clearly discernible in the optical spectra.

**Figure 7:**
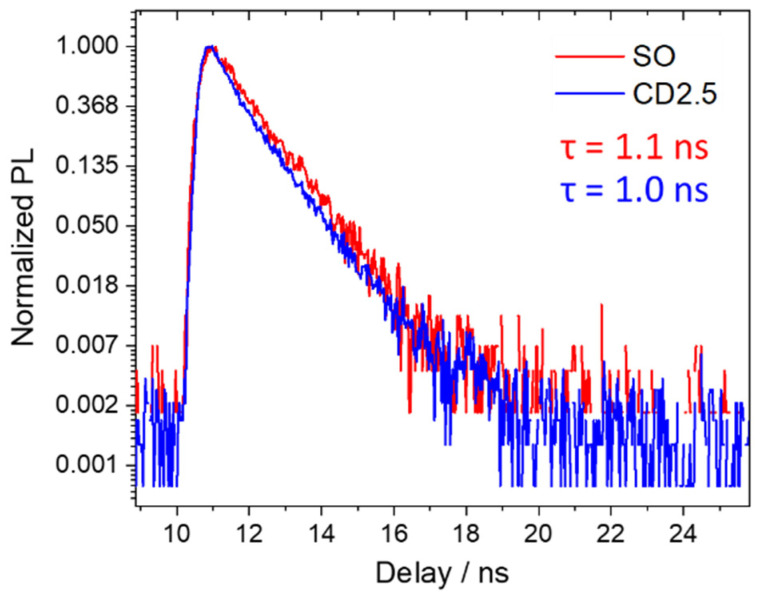
Normalized PL decay profile under excitation at 520 nm in SO and CD2.5.

The red emission PLQY of the obtained CD2.5 under excitation at 532 nm is 4%, which is smaller than the SO QY value (16%). This reduction in QY is most likely due to the contribution of the non-emissive core to the overall optical absorption. Apart from this effect, the SO dye structure and its stability are maintained even after the formation of a carbonaceous shell which, eventually, does not significantly affect the radiative and non-radiative recombination of the embedded dye. Figure 7 displays the time-resolved photoluminescence spectra that confirm the incorporation in the carbon dots matrix. Indeed, the lifetimes of SO and CD2.5 are very close when analyzed with a single exponential decay law (τ_SO_ = 1.1 and τ_CD2.5_ = 1.0 ns), although CD2.5 lifetime results are slightly affected by the matrix environment.

**Figure 8:**
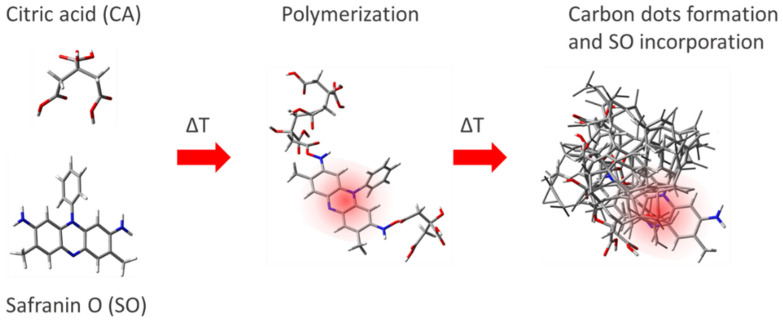
Incorporation of SO in the carbonaceous matrix to form a red-emissive C-dot.

Experimental data on the structure and composition of C-dots (DTA-TGA, FTIR, and XPS), together with the photophysical properties, suggest that the synthesis preserves the red emission of safranin. When safranin is added to dissolved citric acid, it reacts forming a direct amide bond. As the reaction proceeds, citric acid polymerizes and thermally degrades, forming a carbonaceous structure. SO remains incorporated in this structure without losing its characteristic emission (Figure 8).

**Figure 9:**
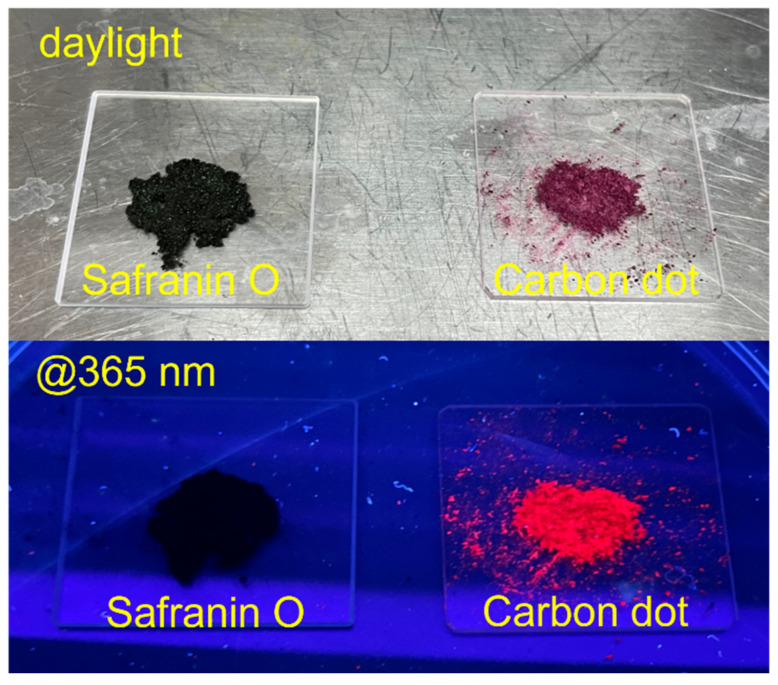
Optical appearance under daylight and solid-state emission (λ_ex_ = 365 nm) of safranin O powder and safranin-based C-dots.

Embedding SO into the C-dot structures gives several advantages, in terms of optical properties, compared with the isolated dye. As shown in Figure 9, the incorporation into a carbonaceous matrix enables the solid-state fluorescence of the safranin-based C-dots (at 2.5 min), which is not detected in the safranin O dye powders. Although slightly lower than in liquid phase, the measured C-dot QY in solid state is remarkably high (2.2%, as measured in an integrating sphere). The QY value suggests that the dot matrix hampers the formation of non-fluorescent aggregates that would occur through π–π interactions between different SO units. The matrix preserves the dye molecules from non-radiative recombination pathways. In contrast, the emission observed from SO in powder is virtually zero (Figure 9). The decay lifetime of the CD2.5 in solid state is very close to that of the same dots dissolved in a liquid phase (Figure S5), confirming that embedding SO into the matrix does not activate new strong non-radiative channels. The moderate reduction of QY from liquid to solid state can be explained considering the partial emission reabsorption, as also suggested by the red shift of the solid-state emission, peaking at 670 nm (Figure S6).

**Figure 10:**
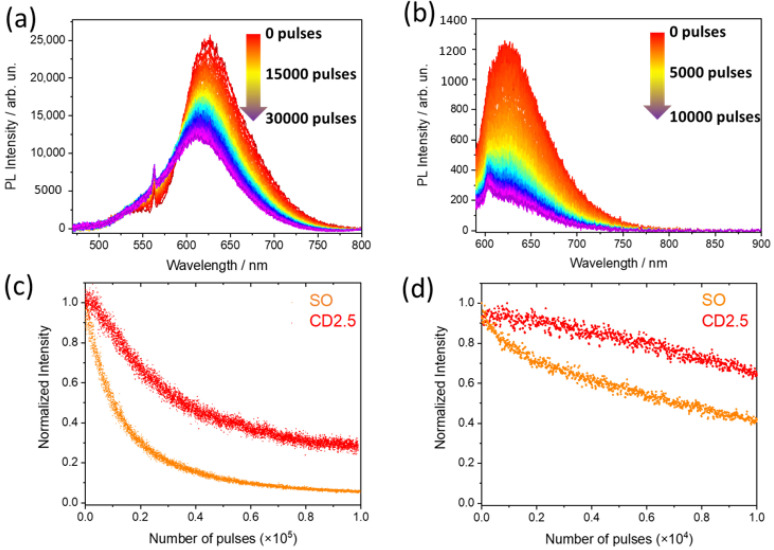
Spectrally resolved photoluminescence of SO as a function of laser pulses under prolonged irradiation, (**a**) λ_ex_ = 532 nm and (**b**) λ_ex_ = 260 nm. Photobleaching kinetics of SO (orange dots) and CD2.5 (red dots) under λ_ex_ = 532 nm (**c**) and λ_ex_ = 260 nm (**d**).

The photochemical stability is one of the most important properties to test for the applications in optics and photonics of fluorescent C-dots. To evaluate the matrix effect on the emission properties, photobleaching experiments have been performed by comparing SO in solution and in the C-dots. Figure 10a,b show the PL spectra change of pure SO, measured under irradiation with high-intensity visible (532 nm, 10 mJ/pulse) and UV (260 nm, 0.15 mJ/pulse) pulsed laser excitation beams. The PL spectra show one emission peaking around 620 nm under both irradiation sources. The peak intensity is observed to decrease by a factor of ~5 after 30,000 pulses under the visible excitation beam (Figure 10a) and by a factor of ~2 after 100,000 pulses under the UV excitation beam (Figure 10b). The observed intensity changes of the SO PL spectra are the results of the gradual bleaching of the photoluminescence, due to the photodegradation of SO under prolonged irradiation by the high-intensity laser beam. A similar experiment has been carried out for comparison on CD2.5 (data not shown) in order to compare the resistance to photobleaching of the two samples.

Figure 10c,d shows the normalized PL kinetics, obtained by analyzing both the CD2.5 and SO spectra variations under the bleaching beams. In both cases, CD2.5 has a better photostability compared with the SO dye. By fitting with single or double exponentials the kinetics (λ_ex_ = 532 nm) (Figure 10c), a photobleaching rate for SO and for CD2.5 of ~[1.3 × 10^4^ pls]^−1^ + [7.5 × 10^4^ pls]^−1^ (double exponential) and ~[3.03 10^4^ pls]^−1^ was measured, respectively. These values correspond to a half-life of 1.1 × 10^4^ and 3.5 × 10^4^ pls for SO and CD2.5, respectively. Thus, the photobleaching rate is slower in CD2.5 by a factor of 3, as compared with the isolated SO dye. Similarly, with the excitation beam at 260 nm, the photobleaching rates obtained for SO and CD2.5 are ~[700 pls]^−1^ + [1.5 × 10^4^ pls]^−1^ (double exponential) and ~[2.7 × 10^4^ pls]^−1^, respectively. The corresponding half-lives are 0.8 × 10^4^ and >10^4^ pulses. The photobleaching experiments shown in Figure 10 have been carried out using very different laser intensities in the UV (260 nm, 0.15 mJ/pulse) and visible range (10 mJ/pulse). For the sake of comparison, a third FLIP experiment was performed using a laser beam with wavelength λ_ex_ = 532 nm and power 0.15 mJ/pulse, but no fluorescence loss was detected (data not shown). This suggests that UV light (260 nm) is inherently more effective than visible light (532 nm) in photobleaching the fluorophores, both in free form and attached to the carbon core. In any case, these results confirm that the carbonaceous matrix prevents dye degradation and protects the molecules from photobleaching.

## 4. Conclusions

The synthesis used in this work overcomes one of the intrinsic limits associated with the synthesis of C-dots. An accurate design of the fluorescence properties, including the characteristic emission, requires a careful control of the structure–properties relationships. In many cases, in fact, the fluorescence originates from the various reactions occurring during the pyrolysis of the precursors. The by-products form families of different fluorescent molecules or polymers. The present synthesis gives a nanoparticle with an emission similar to the precursor fluorophore. This is achieved using a molecule with well-defined optical properties and avoiding its degradation during the preparation of the dot.

The current strategy used to synthesize the C-dots emitting in the red has a general value since it allows the controlling of the structure and origin of the fluorescence. The optically active molecule that is inserted into the carbonaceous matrix is, in fact, at the ground of the dot emission. The matrix itself does not emit, at least in the present synthesis conditions, and has a passive role, i.e., to protect the molecule from photobleaching and self-quenching.

If properly optimized, the strategy of incorporating fluorophores into carbon nanoparticles would contribute to extending the photostability of organic molecules while preserving the characteristic emission of fluorescent dyes.

## Data Availability

Original data are available upon request to the corresponding author.

## Supplementary Materials

The following supporting information can be downloaded at: https://www.mdpi.com/article/10.3390/nano12142351/s1, Table S1: Analysis of C1s and N1s XPS spectra for CA, SO, and C-dots at different reaction times; Figure S1: TGA profiles of CA, SO, and CD2.5 (2.5 min) (left); DSC profiles of CA, SO, and CA + SO (right); Figure S2: Normalized FTIR spectra of CD0 CD2.5 and CD15 in the range between 1500 and 1800 cm^−1^; Figure S3: X-Ray photoelectron spectroscopy analysis of CA and SO (C1s and N1s photoemission spectra); Figure S4: 3D fluorescence spectra (emission (x-scale), excitation (y-scale), intensity (false colour scale)) of SO in solution at a concentration of 0.01 mg/mL; Figure S5: Normalized PL intensity decay under excitation at 532 nm CD2.5 in solution and in solid state at 365 nm; Figure S6: PL spectrum of solid-state CD2.5 acquired with excitation of λ = 365 nm.
