# Supplementary material for: Harnessing Molecular Fluorophores in the Carbon Dots Matrix: The Case of Safranin O
Source: Nanomaterials (Basel). 2022 Jul 9;12(14):2351. doi: 10.3390/nano12142351 (PMC9315549; doi:10.3390/nano12142351)
Supplement: Supplementary file 1 [file nanomaterials-12-02351-s001.zip › nanomaterials-1779759-supplementary.pdf]

nanomaterials

*Supplementary Materials*

# Harnessing Molecular Fluorophores in the Carbon Dots Matrix: The Case of Safranin O

**Manuela Meloni [1], Luigi Stagi [2], Davide Sanna [1], Sebastiano Garroni [2], Laura Calvillo [3], Angela Terracina [4], Marco Cannas [4], Fabrizio Messina [4], Carlo Maria Carbonaro [5], Plinio Innocenzi [1,*] and Luca Malfatti [1,*]**

[1] Laboratory of Materials Science and Nanotechnology (LMNT), Department of Biomedical Sciences, CR-INSTM, University of Sassari, 07100 Sassari, Italy; manmeloni@uniss.it (M.M.); dvdsanna@uniss.it (D.S.)
[2] Department of Chemistry, Physics, Mathematics and Natural Sciences, University of Sassari, 07100 Sassari, Italy; lstagi@uniss.it (L.S.); sgarroni@uniss.it (S.G.)
[3] Department of Chemical Sciences, University of Padua, 35131 Padua, Italy; laura.calvillolamana@unipd.it
[4] Department of Physics and Chemistry Emilio Segrè, University of Palermo, 90123 Palermo, Italy; angela.terracina@unipa.it (A.T.); marco.cannas@unipa.it (M.C.); fabrizio.messina@unipa.it (F.M.)
[5] Department of Physics, University of Cagliari, Cittadella Universitaria, 09042 Monserrato, Italy; cm.carbonaro@dsf.unica.it
* Correspondence: plinio@uniss.it (P.I.), luca.malfatti@uniss.it (L.M.)

**Table S1.** Analysis of C1s and N1s XPS spectra for CA, SO and CDs at different reaction times.

## C 1s

| Samples | C sp$^2$ | C sp$^3$/C–N | C–OH | O=C–NH | O=C–OH |
|---|---|---|---|---|---|
| **CA** | | 284.9 eV **47.0 %** | 285.6 eV **16.3 %** | | 289.0 eV **36.7 %** |
| **SO** | 284.7 eV **70.7 %** | 285.7 eV **29.3 %** | | | |
| **CD0 (0 min)** | 284.7 eV **36.2%** | 285.6 eV **36.2 %** | 286.7 eV **14.8%** | 287.8 eV **4.1 %** | 289.0 eV **8.7 %** |
| **CD2.5 (2.5 min)** | 284.6 eV **52.4%** | 285.6 eV **22.8%** | 286.6 eV **12.3%** | 287.8 eV **4.1 %** | 289.0 eV **5.7 %** |
| **CD15 (15 min)** | 284.7 eV **57.7 %** | 285.6 eV **19.6 %** | 286.6 eV **4.6%** | 288.0 eV **11.2 %** | 288.9eV **6.9 %** |

## N 1s

| Samples | C=N–C | C–NH$_2$ | C$_2$–NH | C$_3$–N |
|---|---|---|---|---|
| **SO** | 398.8 eV **30.6 %** | 399.6 eV **45.5 %** | | 401.0 eV **23.9 %** |
| **CD0 (0 min)** | 397.8 eV **23.9 %** | 399.6 eV **20.1 %** | 400.2 eV **20.9 %** | 401.1 eV **15.7 %** |
| **CD2.5 (2.5 min)** | 398.7 eV **39.5 %** | 399.6 eV **26.6 %** | 400.2 eV **23.4 %** | 401.1 eV **10.5 %** |
| **CD15 (15 min)** | 398.6 eV **18.4 %** | 399.6 eV **39.1 %** | 400.3 eV **25.6 %** | 401.0 eV **16.9 %** |

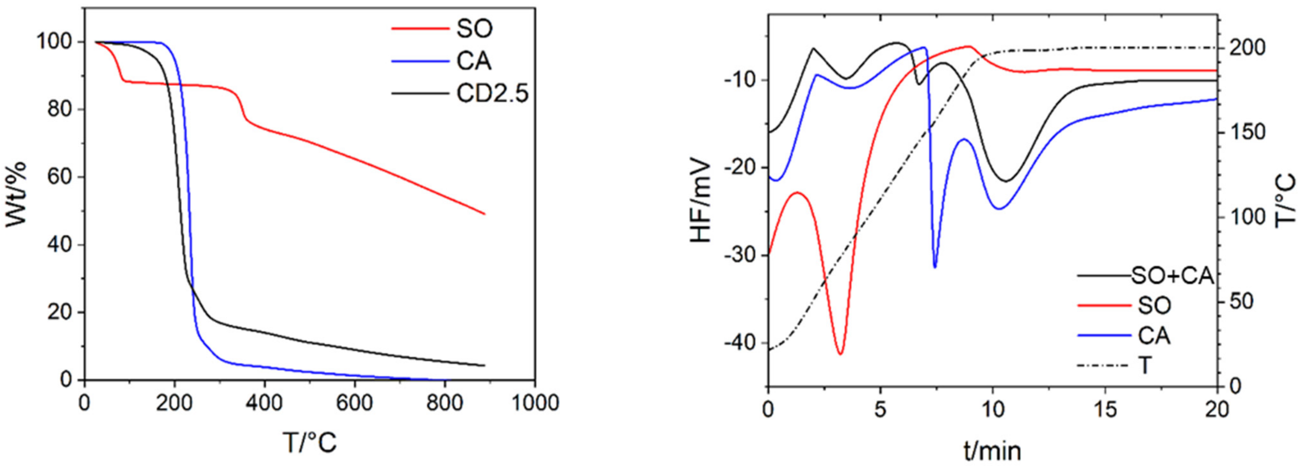


**Figure S1.** TGA profiles of CA, SO, and CD2.5 (2.5 minutes) (**left**); DSC profiles of CA, SO, and a combination of the two reagents (SO + CA) (**right**).

Figure S1 shows on the left the TGA (left) of CA, SO precursors and CD2.5 sample in nitrogen atmosphere from 25 to 900 °C (performed with SDT Q 600 TA INSTRUMENTS). In the TGA profile of the CA is present the typical weight loss step (220 °C) due to polycondensation process, with $H_2O$ loss, and a second step with a smaller weight loss (~10%) around 250°C due to carbonization with release of CO and $CO_2$ gases. In the SO curve, we observe at first an initial weight loss, probably due to residual solvent evaporation at 60°C (SO dye content around 97% mol/mol). After this step, the dye remains stable up to 300 °C where a first thermal degradation is identified. The total weight loss of theCD2.5 sample does not reach the value of 0% at 900 °C, such as for CA, suggesting the presence of high temperature stable carbon compounds that include SO dye degraded species.
The chemical reaction occurring during the thermal synthesis have been studied by DSC (Figure S1 right) using an heating ramp from 25 to 200 °C with a rate of 20°C $min^{-1}$. The final temperature was kept for 10 minutes to simulate the synthesis conditions, and the heat flow variation was evaluated as a function of the reaction time.
The DSC profiles for the pure compounds of CA, SO, and CA + SO combination are in agreement with the TGA findings. The profile of the CA shows three endothermic events: a first one due to a slight loss of water; a second one relative to its melting temperature (around 150 °C, t = 7.5 min) and a third event related to both polycondensation and carbonization process. The SO curve shows, as in the TGA profile, an endothermic peak at about 60 °C attributed to residual solvent evaporation. Finally, SO-CA also shows 3 endothermic peaks: the first due to CA water loss; the second related to the CA melting temperature at 140 °C, lower than the pure citric acid, and finally, the last one at 200 °C associated to the carbonization/degradation of the polymeric matrix. the second endothermic peak is attributed to the polymerization of CA since it is not correlated with a weight loss.

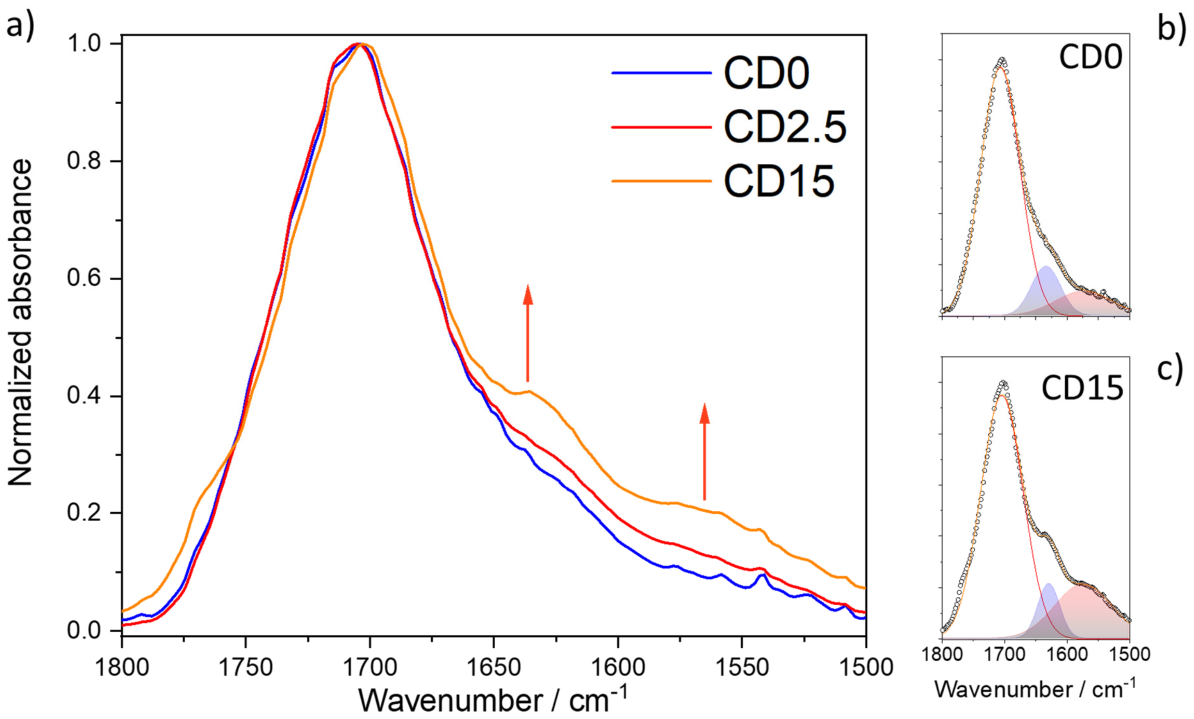


**Figure S2.** (**a**) Normalized FTIR spectra in the range between 1500 and 1800 cm$^{-1}$. (**b**) and (**c**) deconvolution analysis of the three main bands at 1703 cm$^{-1}$, 1636 (C=O; amide I) and 1561 cm$^{-1}$ (NH; amide II) of CD0 and CD15.

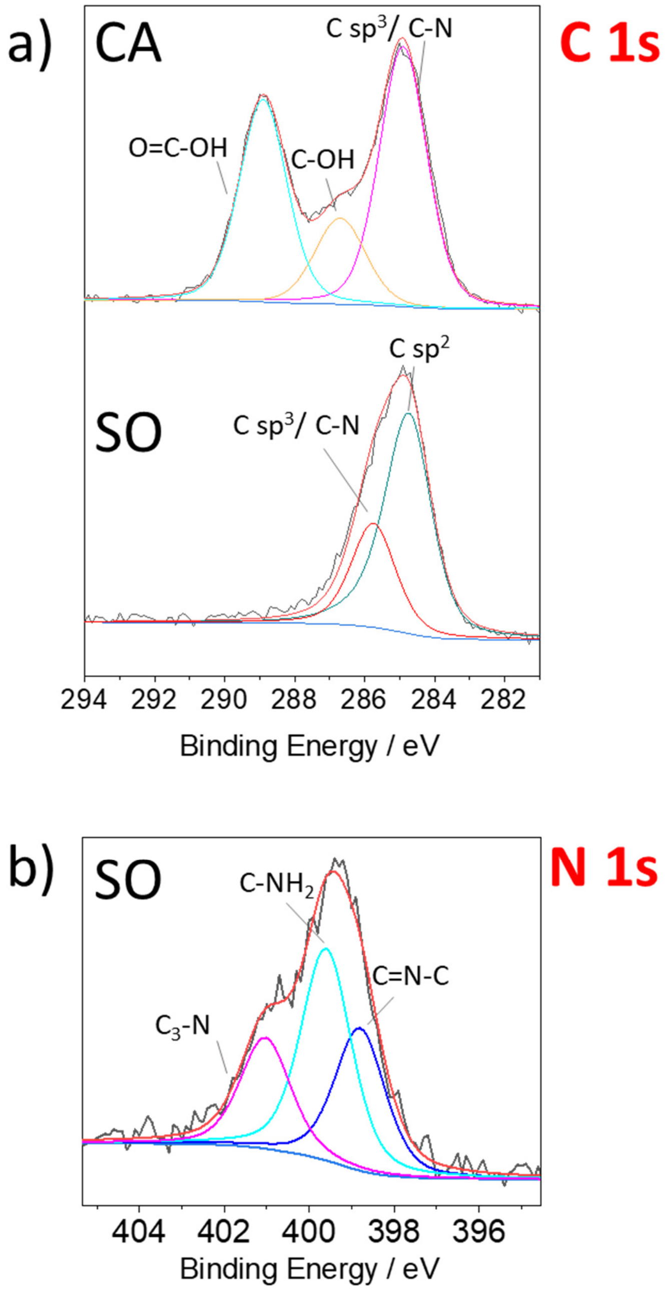


**Figure S3.** X-Ray photoelectron spectroscopy (XPS) analysis of CA and SO (C1s (**a**) and N1s (**b**) photoemission spectra).

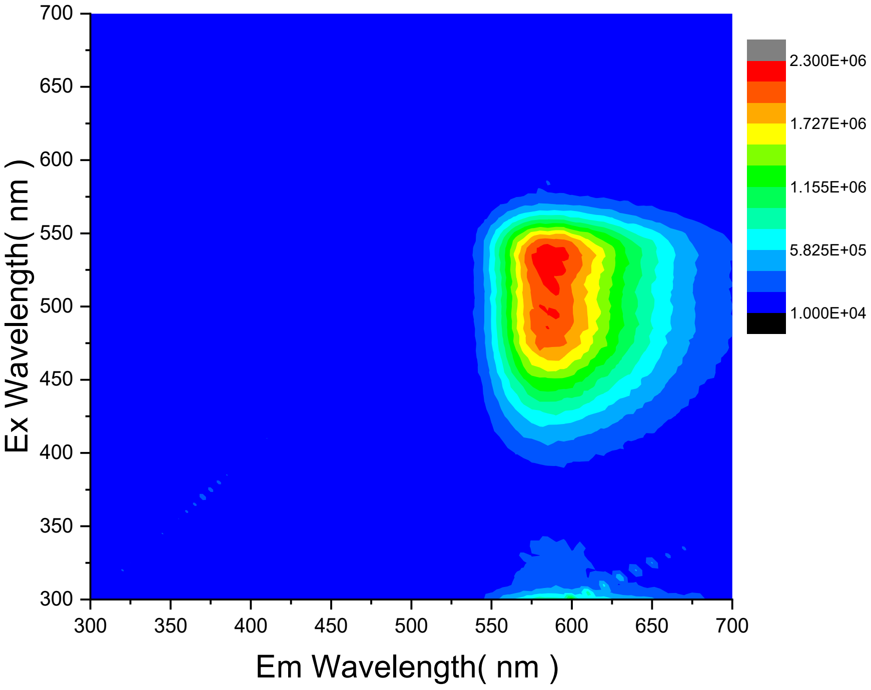


**Figure S4.** 3D Fluorescence spectra (emission (x-scale), excitation (y-scale), intensity (false colour scale)) of SO in solution at a concentration of 0.01 mg $mL^{-1}$.

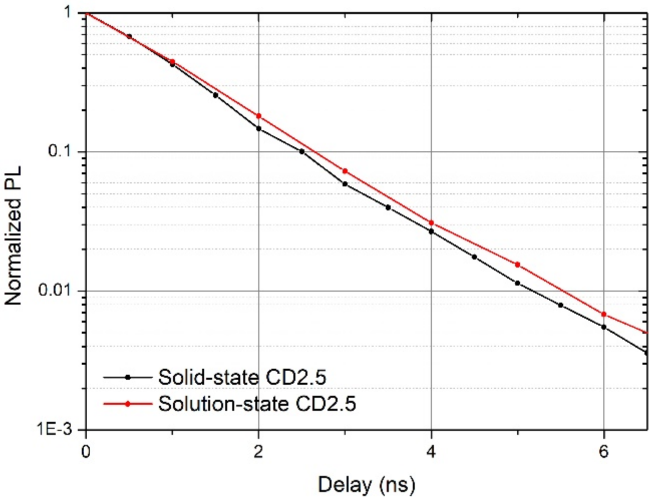


**Figure S5.** Normalized PL intensity decay under excitation at 532 nm CD2.5 in solution and in solid-state at 365 nm.

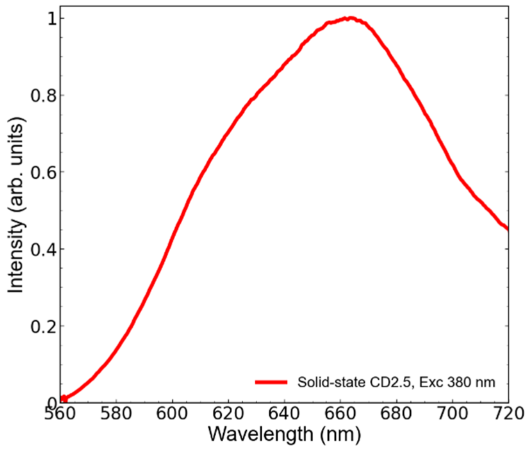


**Figure S6.** PL spectrum of solid-state CD2.5 acquired with excitation of λ=365 nm.
